# Long-term clinical stabilization of scleroderma patients treated with a chronic and intensive IV iloprost regimen

**DOI:** 10.1007/s00296-016-3582-4

**Published:** 2016-10-28

**Authors:** Rosario Foti, Elisa Visalli, Giorgio Amato, Alessia Benenati, Giovanni Converso, Alberto Farina, Salvatore Bellofiore, Massimiliano Mulè, Marcella Di Gangi

**Affiliations:** 1 Rheumatology Unit, A.O.U. Policlinico Vittorio Emanuele, Catania, Italy; 2 Medical Affairs Department, Italfarmaco S.p.A., Milan, Italy; 3 Division of General Thoracic Surgery, A.O.U. Policlinico Vittorio Emanuele, Catania, Italy; 4 Division of Cardiology, A.O.U. Policlinico Vittorio Emanuele, Catania, Italy

**Keywords:** Raynaud’s phenomenon, Scleroderma, Iloprost, Systolic pulmonary arterial pressure, Digital ulcers

## Abstract

Intravenous iloprost is a first-line option for the treatment of scleroderma-related digital vasculopathy, and some studies have suggested its favourable role on disease progression. The aim of our study is to evaluate the disease progression, specifically in terms of cardiopulmonary function, in a group of consecutive patients chronically treated with intravenous iloprost. Our retrospective study enrolled 68 scleroderma patients (68 F, 54.4 ± 12.3 years) treated with iloprost for 7.1 ± 2.9 years, with a schedule of 5–6 consecutive daily infusions per month (6 h/day, 0.5–2.0 ng/kg/min). In all patients, modified Rodnan skin score (4.7 ± 5.3 vs. 3.7 ± 5.3, *p* < 0.0001), systolic pulmonary arterial pressure (sPAP) (30.9 ± 6.4 vs. 24.0 ± 3.2 mmHg, *p* < 0.0001), tricuspid annular plane systolic excursion (22.1 ± 2.4 vs. 23.8 ± 3.5 mm, *p* = 0.0001), pro-brain natriuretic peptide (97.2 ± 69.3 vs. 65.8 ± 31.7 pg/ml, *p* = 0.0005) showed statistically significant improvement from baseline. In the subgroup of patients with baseline sPAP ≥36 mmHg (*n* = 17), a significant sPAP reduction was observed (from 39.5 ± 3.8 to 25.1 ± 4.5 mmHg, *p* < 0.0001) after 7.6 ± 2.5 years of follow-up. The number of patients with digital ulcers (DUs) at follow-up was reduced from baseline (42.6 vs. 11.8%, *p* < 0.001), and none of the free-DU patients at baseline presented DUs at follow-up. An intensive and chronic regimen of IV iloprost administration seems to stabilize and potentially improve the long-term development of disease in SSc patients, as suggested by stabilization or significant improvement of cardiopulmonary parameters and vasculopathy.

## Introduction

Scleroderma (systemic sclerosis or SSc) is a severe, chronic disease characterized by small vessel vasculopathy, autoantibodies production, and fibroblast dysfunction leading to an excessive deposition of collagen in the skin and internal organs [1, 2].

Severe Raynaud’s phenomenon (RP) is the early onset symptom in most SSc patients and may precede other clinical manifestations of the disease by many years [3]. The clinical course of the disease often involves the cardiovascular and respiratory systems, which can be severely damaged by SSc. The heart can be directly or indirectly involved, with myocardial damage, or with the involvement of other organs, especially kidneys and lungs, respectively [4]. For the respiratory system, SSc can affect lung parenchyma and pulmonary blood vessels, leading to interstitial lung disease (ILD), which may progress to pulmonary arterial hypertension (PAH). The presence of a cardio-pulmonary involvement generally leads to a poor prognosis for the patient [2]. Patients with significant internal organ involvement remain often asymptomatic until the late stages of systemic sclerosis; therefore, routine monitoring for the underlying disease and an intensive medical treatment are essential after the first diagnosis. Despite recent advances in the disease management, systemic sclerosis remains a treatable but not curable disease [2].

In the present paper, we report our experience with intravenous iloprost, a stable prostacyclin analogue indicated for the treatment of secondary RP, which may have a favourable effect on the disease progression. Iloprost shows vasodilating, anti-platelet, cytoprotective, and immuno-modulating properties, with a long-lasting beneficial effect on the microcirculation [5]. The European League Against Rheumatism (EULAR) guidelines recommend iloprost as a first-line drug for the treatment of SSc-related digital vasculopathy in order to reduce the frequency and severity of SSc-RP attacks and to heal active digital ulcers (DUs) in patients with SSc, representing the gold standard in the treatment of active ulcers [6]. Moreover, recent studies have described a favourable disease course in SSc patients regularly treated with iloprost, with a low occurrence of the most severe vascular complications such as PAH, renal crisis, and digital necrosis [7–12].

The aim of the present study is to evaluate the disease progression, specifically in terms of cardiopulmonary function, in a group of consecutive SSc patients treated with iloprost, at the Unit of Rheumatology of Catania’s hospital, Italy.

## Methods

Our retrospective study enrolled 68 SSc patients (68 F, 54.4 ± 12.3 years) treated with iloprost for 7.1 ± 2.9 years, with a schedule of 5–6 consecutive daily infusions per month (6 h/day, 0.5–2.0 ng/kg/min). Patients generally received six iloprost infusions per month, with possible suspensions or reductions during the warmest month of the year, for the treatment of secondary RP. The study was conducted under the Declaration of Helsinki and the current ethical standards. Assessed parameters included the following: modified Rodnan skin score (mRss), systolic pulmonary arterial pressure (sPAP), tricuspid annular plane systolic excursion (TAPSE), diffusing capacity of the lung for carbon monoxide (DLCO), forced vital capacity (FVC), alveolar volume (AV), DLCO/AV, pro-brain natriuretic peptide (pBNP), NYHA class, and presence/absence of digital ulcers.

Descriptive statistics was performed by calculating mean and standard deviation (SD) for continuous variables, and percentage for discrete variables. Statistical comparisons of post-baseline versus baseline data were made using Student’s *t* test for paired data when continuous variables were analysed, and by applying the McNemar test, when categorical variables were considered. Statistical analyses were performed through standard procedures using SAS statistical program, version 9.2 (SAS Institute, Cary, NC, USA).

## Results

The characteristics of the study population are described in Table 1. Patients were followed up for an average of 7.1 years, varying from 1 to 15 years, during which they were regularly treated with intravenous iloprost. The age of onset of Raynaud’s phenomenon corresponds in most cases to the onset of the disease and the diagnosis of scleroderma. The evaluations refer to baseline data—before the beginning of treatment with iloprost—and to the last available follow-up data for each patient.

**Table 1:** Characteristics of the study population

Number of patients	68
Age (years ± SD)	54.4 ± 12.3
Age of onset of Raynaud’s phenomenon ± SD	46.2 ± 13.6
Sex	100% F
BMI ± SD	24.4 ± 4.4
Interstitial lung disease (%)	20.6
Type	
Limited (%)	69.1
Diffuse (%)	25.0
Early (%)	5.9
Pattern	
Early (%)	27.9
Active (%)	51.5
Late (%)	20.6
Antibodies	
ANA (%)	100
ACA (%)	48.5
SCL70 (%)	20.6
Duration of treatment with IV iloprost ± SD	7.1 ± 2.9
Number of IV iloprost infusions per year ± SD	57.8 ± 11.2
Bosentan (%)	48.5
Sildenafil (%)	2.9
Losartan (%)	75.5
Hydroxychloroquine (%)	25.0
Azathioprine (%)	23.5
Mycophenolate (%)	22.1
Cyclosporine (%)	1.5

*BMI* Body Mass Index, *IV* intravenous, *SD* standard deviation

The results show a stabilization of the cardiopulmonary function, since these parameters remained unchanged or were significantly improved (Table 2).

**Table 2:** Changes in skin score and cardiopulmonary function over time

	All patients (*n* = 68)		
	Baseline	Follow-up	*p*
mRss ± SD	4.7 ± 5.3	3.7 ± 5.3	<0.0001
sPAP ± SD, mmHg	30.9 ± 6.4	24.0 ± 3.2	<0.0001
NYHA class ± SD	1.0 ± 0.0	1.0 ± 0.4	1.0
TAPSE ± SD, mm	22.1 ± 2.4	23.8 ± 3.5	0.0001
FVC ± SD, % predicted	107.1 ± 14.5	101.2 ± 21.3	0.0581
DLCO ± SD, % predicted	83.7 ± 13.5	81.4 ± 14.3	0.4121
AV ± SD, % predicted	91.1 ± 13.0	91.3 ± 15.3	0.9855
DLCO/AV ± SD, % predicted	88.5 ± 13.5	91.2 ± 14.0	0.0575
pBNP ± SD, pg/ml	97.2 ± 69.3	65.8 ± 31.7	0.0005

*AV* alveolar volume, *DLCO* diffusing capacity of the lung for carbon monoxide, *FVC* forced vital capacity, *mRss* modified Rodnan skin score, *pBNP* pro-brain natriuretic peptide, *SD* standard deviation, *sPAP* systolic pulmonary arterial pressure, *TAPSE* tricuspid annular plane systolic excursion

A significant sPAP reduction from 39.5 ± 3.8 to 25.1 ± 4.5 mmHg (*p* < 0.0001) was also observed in the subgroup of patients with baseline sPAP ≥36 mmHg (*n* = 17), after an average follow-up of 7.6 ± 2.5 years.

In all patients, mRss (4.7 ± 5.3 vs. 3.7 ± 5.3, *p* < 0.0001), tricuspid annular plane systolic excursion (22.1 ± 2.4 vs. 23.8 ± 3.5 mm, *p* = 0.0001), pro-brain natriuretic peptide (97.2 ± 69.3 vs. 65.8 ± 31.7 pg/ml, *p* = 0.0005) showed a statistically significant improvement from baseline. DLCO, AV, DLCO/VA, FVC, and NYHA class remained unchanged.

Table 3 shows the occurrence of DUs (% of patients with at least 1 DU) at baseline and at follow-up, in the whole population and according to the administered treatment. The results suggest the therapy efficacy both in the treatment of active DUs and in their prevention. In particular, the percentage of patients with DUs at follow-up was reduced from baseline ( *p* < 0.001), and none of the free-DU patients at baseline presented DUs at follow-up. The multivariable regression analysis revealed no significant influence of other administered drugs on the magnitude of sPAP reduction.

**Table 3:** Presence of digital ulcers in the study population

Presence of digital ulcers	Baseline (%)	Follow-up (%)
All patients (*n* = 68, follow-up 7.1 ± 2.9 years)	42.6	11.8
Patients according to treatment		
Iloprost (*n* = 35, follow-up 5.8 ± 2.7 years)	5.7	0.0
Iloprost + bosentan (*n* = 33, follow-up 8.3 ± 2.5 years)	81.8	24.2

## Discussion

The present results show a disease stabilization in SSc patients during a long-term follow-up, as indicated by the improvement or non-worsening of the assessed parameters.

This patient’s cohort showed a low mRss at baseline with a slight but significant reduction during the follow-up. This finding might be relevant, since previous studies showed a skin score worsening related to the disease progression in terms of patient survival [13] or occurrence of scleroderma renal crisis [14].

Our results indicate a stabilization of the cardiopulmonary disease, in a very long-term follow-up, possibly suggesting a favourable effect of the treatment against the development of pulmonary arterial hypertension. These conclusions are first supported by NYHA class non-progression and a significant reduction of systolic pulmonary arterial pressure and brain natriuretic peptide levels.

In particular, the importance of sPAP has been recently focused by the EUSTAR working group, since baseline values ≥36 mmHg were significantly associated with an increased risk of death up to 3-year follow-up [HR 1.44 (1.06, 1.96) vs. baseline sPAP < 36 mmHg], regardless of the presence of pulmonary hypertension assessed by right heart catheterization [15]. In our study, average baseline sPAP of the entire population was 30.9 ± 6.4 and 24.0 ± 3.2 mmHg after 7.1 ± 2.9 years of follow-up. Interestingly, in the subgroup of patients with baseline sPAP ≥ 36 mmHg (*n* = 17), a significant reduction of the sPAP was observed (from 39.5 ± 3.8 to 25.1 ± 4.5 mmHg) after an average follow-up of 7.6 ± 2.5 years, and no deaths were recorded.

In the absence of control groups that did not receive iloprost, we compared our data with those reported in recent literature. A recent study by D’Alto et al. [16] reported a significant worsening of sPAP (from 26.1 ± 6.0 to 28.8 ± 6.3) after a 3-year follow-up in a group of 74 consecutive SSc patients, in which IV iloprost was administered in 19% of cases. In the EUSTAR cohorts, where the treatment with IV iloprost is reported in 15.6% of cases [17], overall survival at 5 years is generally comprised between 80 and 90% [15, 18, 19]. Therefore, our results can be considered encouraging.

We also observed a significant change in brain natriuretic peptide levels, which remained below the upper normal limit of 125 pg/ml according to our clinical practice. BNP is released from myocardium in response to wall stress and induces vasodilatation and natriuresis, and high BNP levels are found in patients with cardiac infarction, congestive heart failure, and pulmonary hypertension. High BNP concentration was found to proportionally increase with the degree of right ventricular dysfunction, and further increases are associated with mortality in established severe primary PAH [20]. Thus, BNP levels represent an important diagnostic marker of early pulmonary artery hypertension in SSc patients [20].

Mean TAPSE values remained > 20 mm during the whole study period, with significant improvement from 22.1 ± 2.4 to 23.8 ± 3.5 mm. According to the current guidelines for the diagnosis and treatment of pulmonary hypertension, TAPSE has a well-established importance for assessing disease severity, stability, and prognosis in PAH patients, with a cut-off value >  20 mm indicating a satisfactory patient status [21]. The importance of this parameter is related to the multiple components of right ventricular function, especially with right ventricular ejection fraction (RVEF), in patients with SSc-PAH. In SSc patients, TAPSE values lower than 19.6 mm suggest a RVEF <40%; the lower is the value, the higher is the frequency of hospitalization [22]. Raw mortality data were also found to be significantly associated with TAPSE in SSc patients: values ≤17 mm prognosticated a nearly fourfold increased risk of death compared to patients with TAPSE > 17 mm. Moreover, when calculated as a continuous variable, a decrease of 1 mm in TAPSE was associated with a 15% increased risk of death [23].

We also observed a stabilization of interstitial lung disease markers, such as DLCO, FVC, VA, and DLCO/VA. This is important for both patient’s functionality and prognosis.

Also, our results have a relevant impact on patient’s quality of life since DUs represent a frequent and severe source of pain and disability in SSc. The administration of an intensive IV iloprost regimen, in combination with bosentan when indicated, improved pre-existing ulcer healing and prevented the occurrence of new DUs, confirming the importance of these two therapeutic options in the management of SSc vasculopathy [6]. Moreover, these data provide useful information for the chronic administration of IV iloprost, since both EULAR [6] and manufacturer’s [24] recommendations do not provide satisfactory instructions for the therapy repetition over time, probably because all main randomized controlled trials have been carried out with a single infusion cycle and a short-term follow-up [25, 26]. Thus, since the administration of a single 5-day IV iloprost infusion cycle was already known to improve RP and heal DUs, the present results indicate that its monthly repetition maintains a favourable healing effect, and reduce the occurrence of new DUs over time.

The present study has some limitations mainly due to the trial design. Being a retrospective analysis of a patient’s database, a control group was not provided, and the number of patients was relatively limited. Thus, our results need to be confirmed in further prospective and possibly controlled trials.

## Conclusions

Scleroderma remains a treatable but not curable disease, characterized by a poor prognosis due to the occurrence of cardiopulmonary complications. Therefore, the long-term disease stabilization represents an important therapeutic goal. Intravenous iloprost may play a role in promoting a favourable disease course during a long-term follow-up. Our results confirm that monthly iloprost infusions for six consecutive days might exert a beneficial healing effect and reduce the occurrence of new DUs as well against cardiopulmonary disease development or worsening in SSc patients.
